# Bacteriocin-mediated interactions within and between coexisting species

**DOI:** 10.1002/ece3.354

**Published:** 2012-09-01

**Authors:** Hadas Hawlena, Farrah Bashey, Curtis M Lively

**Affiliations:** 1 Department of Life Sciences, Ben Gurion University of the NegevBeer-Sheva, 84105, Israel; 2 Department of Biology, Indiana UniversityBloomington, Indiana, 47405-3700, USA

**Keywords:** Bacteriocins, entomopathogenic bacteria, interspecific interactions, intraspecific interactions, recognition mechanisms, *Xenorhabdus* bacteria

## Abstract

Bacteriocins are bacteriocidal toxins released by almost all bacteria. They are thought to have a narrow range of killing, but as bacteriocin-mediated interactions have been rarely studied at biologically relevant scales, whether this narrow range of action falls mostly within or mostly between coexisting species in natural communities is an open question with important ecological and evolutionary implications. In a previous study, we systematically sampled *Xenorhabdus* bacteria along a hillside and found evidence for genotypic variability and bacteriocin-mediated interactions within *Xenorhabdus bovienii* and *X. koppenhoeferi* colonies that were collected only a few meters apart. In contrast, colonies that were isolated from the same soil sample were always genetically similar and showed no inhibitions. Here, we conducted pairwise growth-inhibition assays within and between seven *X. bovienii* and five *X. koppenhoeferi* colonies that were isolated from different soil samples; all seven *X. bovienii* colonies and at least three of the *X. koppenhoeferi* have been distinguished as distinct genotypes based on coarse-grain genomic markers. We found signatures for both conspecific and heterospecific bacteriocin inhibitions in this natural community of *Xenorhabdus* bacteria, but intraspecific inhibitions were significantly more common than interspecific inhibitions. These results suggest that bacteriocins have a major role in intraspecific competition in nature, but also suggest that bacterocins are important in mediating interspecific interactions among coexisting species in natural communities.

## Introduction

Bacteriocins – bacteriocidal toxins produced by almost all bacteria – are the most abundant and diverse group of bacterial defense systems (e.g., Riley and Wertz 2002; Riley and Chavan 2007). They differ from traditional antibiotics, as they have a relatively narrow killing spectrum, which is thought to be restricted to closely related species (Riley and Wertz 2002). However, thus far, most of the empirical evidence for bacteriocin-mediated interactions have been documented from inhibition assays among strains that were isolated from a variety of host species and locations against indicator lab strains (e.g., Booth et al. 1977; Gaston et al. 1989; Farias et al. 1992; Gordon et al. 1998, 2007; Lima et al. 2002; Riley et al. 2003; Nigutova et al. 2005; Lux et al. 2007; Nes et al. 2007). Hence, the range of action between coexisting species in natural communities is an open problem with important ecological and evolutionary implications.

A broad strand of theoretical work has explored the role of bacteriocins in maintaining microbial diversity and investigated the evolution of bacteriocin production (Gardner et al. 2004; Kerr 2007). These theoretical studies focus on bacteriocin production and sensitivity as the key difference among strains, where “all else is equal,” and thus can be viewed as models of intraspecific evolution. The documented examples of bacteriocin-mediated interactions also mostly concern intra rather than interspecific interactions (e.g., Riley and Wertz 2002; Riley and Chavan 2007; Hawlena et al. 2010a,b). This is because different genotypes of the same species are more likely to occupy the same ecological niche, and hence undergo more intense competition than different species (e.g., Tilman 1982; Aarssen 1983; Goldberg and Barton 1992). However, what if two species occupy the same niche? Would we expect a higher or a lower abundance of bacteriocin-mediated intraspecific interactions compared with interspecific interactions?

Here, we compared the relative abundance of intraspecific and interspecific bacteriocin-mediated competition in a natural community of bacteria at biologically relevant scales. Classical competition theory predicts that intraspecific competition should be greater than interspecific competition, because individuals of the same species share similar resource requirements (e.g., Tilman 1982; Aarssen 1983; Goldberg and Barton 1992). Bacteriocin-based competition, in addition, involves recognition mechanisms between the actor and recipient genotypes that play an important role in determining the outcome of the interaction. Given the specificity of bacteriocin recognition receptor and translocation systems, it is assumed that bacteriocins are effective on only a small range of targets. It is thus reasonable to assume that different species will have more divergent receptors and translocation mechanisms, resulting in a higher probability of inhibitions between conspecifics relative to heterospecifics (Riley et al. 2003). On the other hand, higher genetic similarity among conspecifics in the bacteriocin itself is assumed to result in shared immunity and lower probability of inhibitions relative to heterospecifics (Gordon and O'Brien 2006). Moreover, more frequent interactions among conspecifics might lead them to evolve greater resistance to bacteriocins, also lowering the probability of conspecific inhibitions.

**Figure 1:**
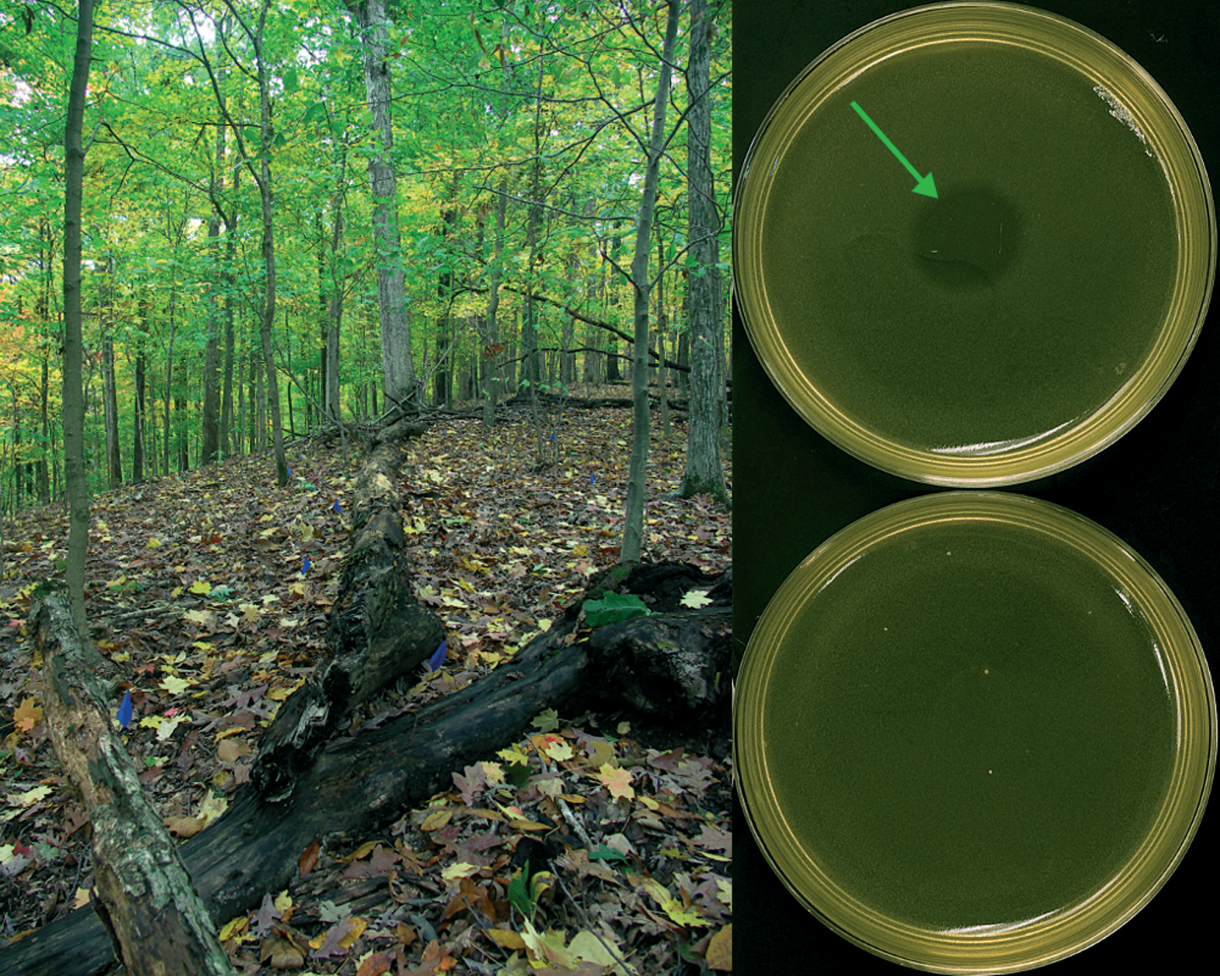
Left: A hillside at Indiana University Research and Teaching Preserve, Moore's Creek, Monroe County, Indiana that was systematically surveyed for *Xenorhabdus* bacteria. Right: Examples of inhibition assays between bacterial colonies. Top photo shows a standard inhibition; bottom photo shows no inhibition.

**Figure 2:**
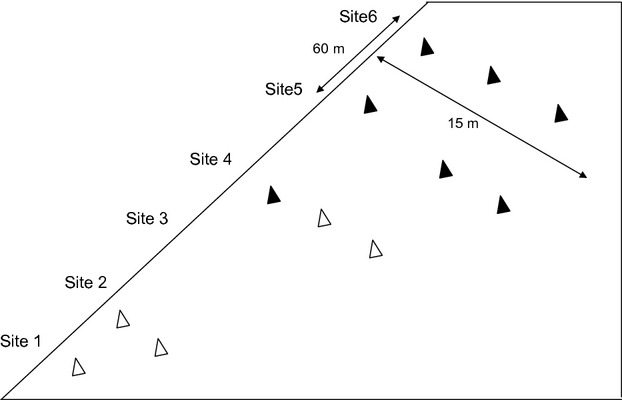
Schematic representation of seven *Xenorhabdus bovienii* (filled triangles) and five *X. koppenhoeferi* (open triangles) bacteria isolated at five sites across a hill. Reproduced from Hawlena et al. (2010a).

*Xenorhabdus bovienii* and *X. koppenhoeferi* are co-occurring insect-killing bacterial symbionts carried by entomopathogenic nematodes in the genus *Steinernema* (Bashey et al. 2011). In a previous study, we systematically sampled *Xenorhabdus* bacteria along a hill at the Indiana University Research and Teaching Preserve (Fig. 1, left) and found a divergent distribution of these two bacterial species (Hawlena et al. 2010a): *X. bovienii* bacteria were isolated from sites located uphill, and *X. koppenhoeferi* bacteria were isolated from sites located downhill, with a small overlap between them (Fig. 2). The two species carry genes that encode xenorhabdicin, a phage-tail-like particle similar to *Pseudomonas* R-type pyocins (Bashey and Forst, unpublished data). Evidence suggests that xenorhabdicin-mediated interactions occur within each *Xenorhabdus* species between colonies that were isolated only few meters apart (Hawlena et al. 2010a,b), and that these interactions successfully predict the fate of intraspecific competition within the insect host (Bashey et al. 2012). Moreover, there is evidence from a laboratory strain of *Xenorhabdus* that xenorhabdicin can play a key role in interspecific interactions (Morales-Soto and Forst 2011). However, the relative importance of intra versus interspecific bacteriocin-mediated interactions in natural communities at these local scales, and whether bacteriocin-mediated interactions can explain the divergent distribution of the two *Xenorhabdus* species remains to be determined.

Of the 20 *X. koppenhoeferi* and the 34 *X. bovienii* isolates that were phenotypically and genetically characterized over the preserve hill, only colonies that were isolated from different soil samples have shown intraspecific variability (Hawlena et al. 2010a,b). Here, we determined the frequency of intraspecific versus interspecific bacteriocin-mediated competition, using seven *X. bovienii* and five *X. koppenhoeferi* natural isolates that were collected from soil samples meters apart; all the *X. bovienii* colonies and at least three of the *X. koppenhoeferi* were distinguished as distinct genotypes based on a coarse-grain, genomic fingerprinting analyses (Hawlena et al. 2010a,b). We conducted pairwise growth-inhibition assays within and between *Xenorhabdus* species. A higher proportion of intraspecific than interspecific bacteriocin-mediated interactions was found, suggesting that a major role of bacteriocins in nature is to mediate intraspecific microbial dynamics. The signatures of both intraspecific and interspecific bacteriocin-mediated interactions suggest that bacteriocins play an important role in mediating bacterial community interactions in nature at local scales.

## Methods

### Bacteria isolates and inhibition assays

The seven isolates of *X. bovienii* and five isolates of *X. koppenhoeferi* used in this study were obtained from separate soil samples collected in 2007 as described in Hawlena et al. (2010a,b) from Indiana University Research and Teaching Preserve, Moore's Creek, Monroe County, Indiana (Fig. 2). Briefly, each soil sample was baited with a larva of the greater wax moth (*Galleria mellonella*), and a single bacterial colony was isolated from nematodes emerging from each caterpillar carcass. Each bacterial colony was described to species by sequencing of the 16S rRNA gene and identified as a distinct genomic fingerprint type by banding patterns based on PCR amplification of enterobacterial repetitive intergenic consensus sequences (Tailliez et al. 2006; Hawlena et al. 2010b).

We performed 144 growth-inhibition assays to determine the pairwise relationships between colonies belonging to the same species (49 assays for *X. bovienii* and 25 for *X. koppenhoeferi*) and colonies belonging to different species (70 assays of *X. bovienii* vs. *X. koppenhoeferi*). The results of these inhibition assays were found to be robust. First, there were no false positive responses based on 12 self-tests and 38 negative-control tests. In the self-tests, the same isolate was used both as the recipient and as the actor, and no inhibition was observed, as bacteriocin-producing clones carry immunity to their own toxin (Riley and Chavan 2007). In the negative-control tests, we tested each isolate's response as a recipient by applying a supernatant that was produced at the same time and according to the same protocol (see below), but without the addition of bacteria. Second, there were no false negative responses based on 32 positive-control tests. The positive-control tests were performed between two strains that had shown repeatable inhibitions in a prior study at the same time and according to the same protocol that was applied in the other growth-inhibition assays.

The release of bacteriocins has been demonstrated to be a major factor affecting the outcome of intraspecific and interspecific competitive interactions within the insect host (Morales-Soto and Forst 2011; Bashey et al. 2012). Our goal was to compare the frequency of antagonistic weapons that kill conspecifics with those that kill heterospecifics, and thus we chose to induce toxins, and not to conduct competition trials. By the use of induction, we controlled for possible biotic and abiotic factors (e.g., competitor density, temperature) that may affect the rate of toxin release. We employed a modified version of the Pugsley and Oudega (1987) method (which uses mitomycin C) to induce the production of bacteriocins in each field isolate. We chose this method over induction by heat or induction at stationary phase of the cultures as it: (1) detects the largest number of bacteriocin producers (Riley et al. 2003); (2) has the highest repeatability (84–100%; Hawlena, unpublished data); and (3) is the most common induction method employed in growth-inhibition assays (reviewed by Riley and Chavan 2007), thus allowing us to compare the results to other studies.

Cells in log-phase growth were incubated with mitomycin C (0.5 *μ*g/mL) at 28°C. After 5 h, 67 *μ*L of chloroform was added to 1 mL of the induced cultures, which were then centrifuged for 10 min at 10,000 g. We filtered the resulting supernatant through a 0.45 *μ*m HT Tuffryn membrane and stored it at 4°C less than 20 days before use. To test the sensitivity of a clone, molten soft (MS) agar (0.6% agar) was sowed with 2% (v/v) of its stationary-phase liquid culture. Then, 10 *μ*L of supernatant of actor isolates was spotted onto the surface of a MS agar plate containing the potential sensitive clone. Plates were incubated for 48 h, at which time inhibition could be visualized as a clear zone on the recipient lawn (Fig. 1, right). All the actor extracts that killed the recipient cells were confirmed to be bacteriocins rather than bacteriophages based on serial dilutions and freezing trials (Hawlena et al. 2010a,b).

### Statistical analyses

For each pair of field isolates, we gave a score for competitive outcome as follows: 0 if the two isolates did not inhibit each other; 1 if one isolate inhibited the other; and 2 if the two isolates inhibited each other. We also gave a score of the genetic similarity as the proportion of ERIC bands shared by two isolates. We performed a Mantel test to confirm the expectation that genetic relatedness is higher in conspecifics (coded as “0”) compared with heterospecific bacteria (coded as “1”). We also used Mantel test to determine the correlation between the competitive outcome and the type of interaction (intraspecific vs. interspecific competition). In each Mantel test, a negative correlation indicates higher values for the conspecific pairings relative to the heterospecific pairings. All statistical tests were two-tailed.

## Results

**Figure 3:**
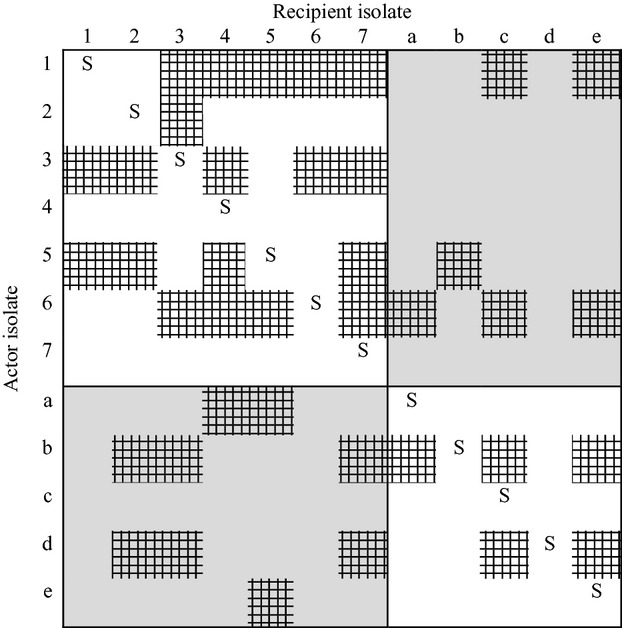
Bacteriocin-mediated competition between pairs of *Xenorhabdus bovienii* isolates (1–7, white background), pairs of *X. koppenhoeferi* isolates (a–e, white background), and between pairs of the two species (gray background). Hatched cells indicate inhibition of recipient (columns) growth by cell-free extracts of actor cultures (rows), whereas empty cells indicate interactions in which the recipient growth was not affected by cell-free extracts of actor cultures. “S” indicates self-tests, in which, in accordance with the theory, no inhibitions were detected.

As expected, we found that the genetic similarity between conspecifics (0.81 ± 0.02) was greater than between heterospecifics (0.40 ± 0.02; *r* = −0.85, *P* < 0.005). Of the 132 growth-inhibition assays (144 excluding 12 self-tests), we observed 39 inhibitions of recipient growth by cell-free extracts of actor cultures. Sixty-two percent of these inhibitions (24/39) were between conspecific pairs, and that was significantly greater than between heterospecific pairs (15/39; *r* = −0.29, *P* < 0.05; Fig. 3).

## Discussion

We found signatures for both conspecific and heterospecific bacteriocin inhibitions in a natural community of *Xenorhabdus* bacteria, but inhibitions were more common in conspecific than in heterospecific pairs. These results suggest that a major role of bacteriocins is to mediate intraspecific competition, but also suggest that bacterocins are important in mediating interspecific interactions among coexisting species in natural communities.

The assumption that bacteriocins predominately kill conspecific strains has served as the basis for much of the experimental and theoretical work exploring the role of bacteriocins in mediating population dynamics (Riley and Chavan 2007). Indeed, most previous studies of gram-negative bacteriocins have described bacteriocins as narrow-spectrum toxins, referring to the observation that they are active against conspecific members, and generally display restricted levels of inhibition outside of the producing species. Nevertheless, bacteriocins have been found to kill across a rather broad phylogenetic range, suggesting that an important role for bacteriocins may be interspecific interactions (Tait and Sutherland 2002; Riley et al. 2003; Bakkal et al. 2010). These contrasting findings raise the question of what is happening in natural communities at local scales in which different genotypes from the same species or from different species may interact.

Here, we provide evidence from a natural bacterial community – a community of *Xenorhabdus* bacteria that coexist over biologically relevant scales – that bacteriocin inhibitions are more common between conspecific than between heterospecific natural isolates. The lower proportion of interspecific inhibitions is not likely to be a result of the greater spatial distances between heterospecific compared with conspecific isolates (Fig. 2), as within each of these bacterial species, the probability of inhibitions increases with spatial distance (Hawlena et al. 2010a,b). Our results thus support the importance of bacteriocins in mediating intraspecific interactions, despite factors such as clonal expansion and the evolution of resistance, which might lessen its role. Indeed, despite their key demonstration of a surprisingly broad phylogenetic killing breadth of bacteriocins, Riley et al. (2003) found that on average bacteriocins are most effective at killing indicator strains from the producer's own species. Crucially, in our study, the inhibition assays were conducted with field isolates that occur in the same ecological niche and reflect the genetic variability found along a hillside. Thus, our study confirms the importance of bacteriocin-mediated intraspecific competition in natural ecological communities. Similar experiments with other microbial groups sharing the same niche are needed to establish the generality of these findings.

The relative strength of intraspecific and interspecific competition has long been investigated (Connell 1983). Classical competition theory predicts that intraspecific competition should be greater than interspecific competition because individuals of the same species share similar resource requirements (e.g., Tilman 1982; Aarssen 1983; Goldberg and Barton 1992). There is not always supportive for this prediction (e.g., Goldberg and Barton 1992; Gurevitch et al. 1992; Hu and Tessier 1995), but in general, in stable environments where there is no stage and age structure, intraspecific competition is mostly greater than interspecific competition (Connell 1983; Wassmuth et al. 2009 and references therein). The main conclusion of these studies is that intraspecific competition plays an important role in species coexistence, as it may self-limit each species to densities that are below the densities necessary to eliminate competing species. Our data suggest that bacteriocin-mediated interactions between conspecific may play a similar role in maintenance of species diversity. Furthermore, the high frequency of bacteriocin-mediated intraspecific interactions may explain the great genetic variability observed within bacterial species (e.g., Vos and Velicer 2006) due to non-transitive interactions between bacteriocin producers, bacteriocin sensitives, and bacteriocin resistant strains (Kerr 2007).

The greater abundance of intraspecific inhibitions compared with interspecific inhibitions may suggest that the divergent distribution of the two *Xenorhabdus* species is not a consequence of bacteriocin-mediated interactions, but is rather determined by other mechanisms such as differences in sensitivity of the two bacterial species to abiotic conditions. Alternatively, the divergent distribution may hint that intensive interspecific inhibitions between the species were common in the past before the species were selected for habitat selectivity (i.e., the ghost of competition past; Connell 1980). Either way, our findings suggest that bacteriocins play an important role in mediating bacterial community interactions in nature at local scales.
